# ATR, a DNA Damage Signaling Kinase, Is Involved in Aluminum Response in Barley

**DOI:** 10.3389/fpls.2019.01299

**Published:** 2019-10-22

**Authors:** Miriam Szurman-Zubrzycka, Malgorzata Nawrot, Janusz Jelonek, Mariusz Dziekanowski, Jolanta Kwasniewska, Iwona Szarejko

**Affiliations:** ^1^Department of Genetics, Faculty of Biology and Environmental Protection, University of Silesia in Katowice, Katowice, Poland; ^2^Department of Plant Anatomy and Cytology, Faculty of Biology and Environmental Protection, University of Silesia in Katowice, Katowice, Poland

**Keywords:** aluminum, ATR, DDR pathway, barley, TILLING

## Abstract

Ataxia Telangiectasia and Rad-3-related protein (ATR) is a DNA damage signaling kinase required for the monitoring of DNA integrity. Together with ATM and SOG1, it is a key player in the transcriptional regulation of DNA damage response (DDR) genes in plants. In this study, we describe the role of ATR in the DDR pathway in barley and the function of the *HvATR* gene in response to DNA damages induced by aluminum toxicity. Aluminum is the third most abundant element in the Earth’s crust. It becomes highly phytotoxic in acidic soils, which comprise more than 50% of arable lands worldwide. At low pH, Al is known to be a genotoxic agent causing DNA damage and cell cycle arrest. We present barley mutants, *hvatr.g* and *hvatr.i*, developed by TILLING strategy. The *hvatr.g* mutant carries a G6054A missense mutation in the *ATR* gene, leading to the substitution of a highly conserved amino acid in the protein (G1015S). The *hvatr.g* mutant showed the impaired DDR pathway. It accumulated DNA damages in the nuclei of root meristem cells when grown in control conditions. Terminal deoxynucleotidyl transferase-mediated dUTP nick-end labeling (TUNEL) analysis revealed that 60% of mutant nuclei possessed DNA nicks and breaks, whereas in the wild type only 2% of the nuclei were TUNEL-positive. The high frequency of DNA damages did not lead to the inhibition of the cell cycle progression, but the mutant showed an increased number of cells in the G2/M phase. In response to treatments with different Al doses, *hvatr.g* showed a high level of tolerance. The retention of root growth, which is the most evident symptom of Al toxicity, was not observed in the mutant, as it was in its parent variety. Furthermore, Al treatment increased the level of DNA damages, but did not affect the mitotic activity and the cell cycle profile in the *hvatr.g* mutant. A similar phenotype was observed for the *hvatr.i* mutant, carrying another missense mutation leading to G903E substitution in the HvATR protein. Our results demonstrate that the impaired mechanism of DNA damage response may lead to aluminum tolerance. They shed a new light on the role of the ATR-dependent DDR pathway in an agronomically important species.

## Introduction

Aluminum (Al) is the most abundant metal and the third most abundant chemical element (after oxygen and silicon) in the Earth’s crust that comprises approximately 8% of its mass (Bojórquez-Quintal et al., 2017). It is a highly reactive element that in a neutral pH is incorporated into various non-toxic minerals, mainly in the form of aluminum oxides and aluminosilicates. In acidic conditions, aluminum solubilizes into highly phytotoxic [Al(H_2_O)_6_]^3+^ molecules, referred to as Al^3+^ cations, which can be easily absorbed by plants (Mossor-Pietraszewska, 2001; Rahman et al., 2018). The toxicity of Al^3+^ in acidic soils is an important agricultural problem that has been identified more than 100 years ago (Miyake, 1916). Acidic lands (pH 5.5 and lower) are spread worldwide—it is assumed that 50% of the world’s potentially arable soils have low pH (Kochian et al., 2015). In tropical and subtropical regions, soil acidity is one of the most important constrains that hinder the increase of food production, but also in the temperate zones of eastern North America and throughout Europe (where acidic soils reach up to 80% of the total area), Al stress may significantly affect crop yields (von Uexküll and Mutert, 1995; Aggarwal et al., 2015). Furthermore, modern farming practices, such as application of ammonium-based fertilizers, as well as industrial pollution unceasingly increase the acidification of soils (Kochian et al., 2005). The first and the most evident symptom and the important effect of aluminum toxicity is the reduction of root growth caused by inhibition of cell divisions in the root tip and decreased elongation of root cells. Additionally, Al^3+^ ions reduce the uptake of water and nutrients and consequently reduce plant growth and yield. Taken together, aluminum toxicity is considered as the main growth-limiting factor in acidic soils and the second, after drought, most serious abiotic stress to crop production worldwide (Kochian et al., 2015).

The best-known mechanism of aluminum tolerance is Al exclusion that is based on the exudation of organic acids (OAs) from the root tip to the rhizosphere. The OA transporters localized in the plasma membrane are activated by the presence of Al^3+^ ions in the environment. The excreted OAs, mainly citrate and/or malate, act as chelators of Al^3+^ ions, forming compounds that do not enter the root and are not toxic to plants. Another mechanism of tolerance, when Al^3+^ cations enter the root cells, is based on the internal formation of OAs and other organic compounds that form complexes with Al. Such complexes are sequestered and detoxified in vacuoles or translocated away from the root tip to the less Al-sensitive parts of the plant (reviewed in Kochian et al., 2015; Riaz et al., 2018).

The mechanisms of Al tolerance involving OAs are well understood; however, the true biochemical targets of Al^3+^ ions and the mechanisms of Al toxicity have not been fully characterized. The primary targets of Al^3+^ in apoplast are negatively charged compounds of the cell wall, such as hemicellulose or pectins (Yang et al., 2011). Aluminum alters the cell wall properties and causes cell wall rigidity, which affects cell elongation. However, Al^3+^ ions may interfere with multiple sites in both symplast and apoplast, and therefore, the exact multilevel molecular mechanisms underlying Al toxicity remain elusive (Singh et al., 2017, Riaz et al., 2018).

Studies carried out on many plant species, including *Arabidopsis* and barley, show that aluminum causes DNA double-strand breaks in root meristem cells, which indicates that DNA is a target for Al^3+^ ions (Nezames et al., 2012; Jaskowiak et al., 2018). Further work performed on *Arabidopsis* has clearly shown that the DNA damage response (DDR) pathway is involved in the Al response (Eekhout et al., 2017). The *Arabidopsis* Al-hypersensitive mutant *als3-1* has been used for suppressor screening to find mutations that can reverse its phenotype. The *als3-1* mutant carries a loss-of-function mutation in the *AtALS3* gene encoding an ABC transporter involved in the translocation of aluminum away from the root tip (Larsen et al., 1997; Larsen et al., 2005). The second-site mutagenesis revealed four suppressor genes: *ATR—Ataxia Telangiectasia and Rad3-related* (Rounds and Larsen, 2008), *ALT2—Aluminum Tolerant 2* (Nezames et al., 2012), *SOG1*—*Suppressor of Gamma response 1* (Sjogren et al., 2015), and *SUV2—Sensitive to UV 2* (Sjogren and Larsen, 2017), all of them involved in the DDR pathway. The mutations identified in these suppressor genes reversed a severe Al hypersensitivity observed in the *als3-1* and increased aluminum tolerance in the wild-type plants (summarized in Eekhout et al., 2017).

In general, in response to the DNA damage, the DDR pathway coordinates a transient cell cycle arrest and DNA repair. ATR is a key cell cycle checkpoint regulator that is required for the monitoring of DNA integrity (Culligan et al., 2004). It is a serine/threonine kinase that, together with Ataxia Telangiectasia Mutated (ATM), transmits the DNA damage signals to the downstream effectors by phosphorylating SOG1 (Rounds and Larsen, 2008). It was shown that ATR is activated when persistent ssDNA is accumulated in the nucleus, whereas ATM is activated in the presence of DSBs (DNA double-strand breaks) (Hu et al., 2016). SOG1, which may be phosphorylated by both ATR and ATM, is a central DDR transcription factor that activates the expression of hundreds of genes involved in DNA repair and cell cycle inhibition (Yoshiyama et al., 2009; Yoshiyama et al., 2013). ALT2 is a WD-40 protein that has, so far, an undefined role; however, it is required for the assessment of DNA integrity, including the monitoring of DNA crosslinks (Nezames et al., 2012). *SUV2* encodes a putative ATRIP (ATR-interacting protein) homologue that co-localizes with ATR and helps in the ATR recruitment to the persistent single-stranded DNA in *Arabidopsis* (Sakamoto et al., 2009; Sweeney et al., 2009). Taken together, the identified genes arrest the cell cycle progression after Al exposure in *Arabidopsis*; thus, their loss-of-function mutations induced in the *als3-1* background led to the progression of cell divisions regardless of the presence of aluminum. The factors encoded by these genes are thought to work together in detecting Al-induced DNA damage. They inhibit the cell cycle progression in order to repair the DNA damage and, eventually, to promote terminal differentiation and endoreduplication (Sjogren et al., 2015; Eekhout et al., 2017).

Due to the fact that soil acidification is a global problem in agriculture, it is important to broaden the knowledge on the mechanisms of Al toxicity in agronomically important crops. Barley (*Hordeum vulgare* L.), which is the fourth cereal crop in regard to cultivation area and production tonnage, is considered as the most Al-sensitive species among the cereals (Wang et al., 2006). In this study, we indicate for the first time the role of ATR in the DDR pathway in barley and the function of the *HvATR* gene in response to Al toxicity. Using TILLING strategy and our *Hor*TILLUS population (Szurman-Zubrzycka et al., 2018), we have developed a barley mutant *hvatr.g* carrying a G6054A missense mutation that leads to the substitution of a highly conserved amino acid in the ATR protein (G1015S). The *hvatr.g* mutant showed an increased tolerance to Al treatment and a high accumulation of DNA damages in root meristem cells, both in control and Al treatment conditions. Despite the accumulation of DNA damages in response to Al, the cell cycle progression was not arrested in the mutant, while the cell cycle profile in root meristems differed between the mutant and its parent. We confirmed the hypothesis on the involvement of *HvATR* in response to DNA damages in barley through the analysis of another mutant in the *HvATR* gene (*hvatr.i*), with a similar to *hvatr.g*, though weaker phenotype. Our results demonstrate that *hvatr* mutants have an impaired mechanism of DNA damage response that leads to aluminum tolerance.

## Material and Methods

### Plant Material

The *Hor*TILLUS (*Hordeum-*TILLING-University of Silesia) population, which is a barley TILLING population developed at the Department of Genetics, University of Silesia in Katowice, has been used as the material for mutational screening in the *HvATR* gene. This population has been created after double treatment of seeds of spring barley cultivar ‘Sebastian’ with sodium azide (NaN_3_) and *N*-methyl-*N*-nitrosourea (MNU) (Szurman-Zubrzycka et al., 2018).

### Mutational Screening of the *HvATR* Gene Using the TILLING Strategy

The whole genome sequence of barley (second version) has been screened in order to find potential homolog(s) of the *Arabidopsis ATR* gene. Based on bioinformatics analysis with the use of Ensembl Plants datasets and tools (http://plants.ensembl.org/index.html), we have identified the putative barley *ATR* gene with acc. no HORVU7Hr1G118750. The gene is located on chromosome 7 and encodes a 2,575-aa protein. The *HvATR* gene has no paralogs in barley genome. The domains and motifs in a putative HvATR protein were computationally predicted with the use of InterProScan (http://www.ebi.ac.uk/interpro/search/sequence-search) and Conserved Domain Search Service at the NCBI website (https://www.ncbi.nlm.nih.gov/Structure/cdd/wrpsb.cgi). Four domains characteristic of ATR proteins were identified: 1) the UME domain of unknown function, 2) the FAT domain, 3) the PIKKc domain that is a phosphatidylinositol 3-/4-kinase catalytic domain, and 4) the FATC domain at the C-terminus. We have selected two conserved fragments of *HvATR* for mutational screening (**Supplementary Material 1**):

1. A 930-bp fragment encoding the UME domain that was proven to be involved in Al response in *Arabidopsis* (Rounds and Larsen, 2008) (T1 amplicon) and
2. A 865-bp fragment encoding a part of the PIKKc domain that includes a kinase catalytic center (T2 amplicon).

The PCR reactions for the T1 and T2 amplicons were optimized for specific primers labeled with IRDye-700 and IRDye-800 (forward and reverse primers, respectively) (**Supplementary Material 2**). Eight-fold DNA pools of M_2_*Hor*TILLUS plants were used as initial templates for mutation detection. TILLING screening was based on Celery Juice Extract (CJE) digestion of heteroduplexes followed by electrophoresis on polyacrylamide gels in the LI-COR DNA sequencers, according to the protocol described elsewhere (Szurman-Zubrzycka et al., 2017; Jost et al., 2019). All putative mutations were confirmed by sequencing.

### Aluminum Treatment in Hydroponics

The system for studying the effect of Al treatments on root growth was based on the method described by Jaskowiak et al. (2018). The hydroponic setup consisted of plastic containers with a capacity of 4.5 L covered with lids containing 12 openings and air distributors with 12 outlets connected to the air pump. The Magnavaca solution was used as a medium for treatment with AlCl_3_ (Magnavaca et al., 1987). The fraction of the bioavailable Al^3+^ ions was calculated using GEOCHEM-EZ software (Shaff et al., 2010). In the presented study, the Al concentration always refers to the bioavailable fraction of Al^3+^ ions. The Al concentrations used in our assays were 0, 5, 10, and 15 μM of bioavailable Al^3+^, which correspond to 0, 25, 50, and 75 μM of nominal AlCl_3_ added to the medium. The pH of the medium was determined each day and maintained at 4.0 throughout the whole experiment.

The seeds of the analyzed genotypes were surface-sterilized in 5% sodium hypochlorite and put on Petri dishes filled with wet filter paper for imbibition at 4°C for 72 h in the dark, and then transferred to 25°C for another 48 h. Afterwards, the seedlings with roots approximately 1.5–4 cm long (depending on the genotype) were implanted into the openings on the container lids, 12 seeds per container, in such a way that their roots were submerged in the medium. The experiments were carried out in a growth room under controlled conditions: light intensity, 250 μM m^−2^ s^−1^; temperature, 20°C/18°C (day/night); and photoperiod, 16/8 h. The seedlings were grown in hydroponics for 7 days. One container was assumed as one biological repetition, with three repetitions per treatment.

### Flood-and-Drain Semi-Hydroponics for Evaluation of Root System Growth

In order to characterize the root system of the *hvatr.g* mutant and its wild-type ‘Sebastian’ grown under optimal conditions, the germinated seeds (prepared as described above) were transferred into a flood-and-drain semi-hydroponic system described by Slota et al. (2016). Briefly, the seedlings were grown individually in acrylic tubes filled with soda lime-glass beads (MEGAN, Poland) and supplemented with a Hoagland medium (Hoagland and Arnon, 1950) through an automatic drip irrigation system. The medium was delivered to the tubes every 15 min through an afferent pump controlled remotely by a programmable logic controller. The acrylic tubes had a bottom drainage opening to ensure draining of the medium. The experiment was carried out for 14 days under conditions of a growth room described above. The experiment was conducted in four repetitions with 10 plants per one biological repetition.

### Root System Scanning and Image Analysis

After 7 days of the Al hydroponic experiment or 14 days of the flood-and-drain root system experiment, seedlings were preserved in 50% ethyl alcohol and their root systems were scanned in waterproof trays filled with water using a specialized scanner with a dual-lens system (EPSON PERFECTION V700 PHOTO) and a WinRHIZO software (Regent Instruments). The root parameters were evaluated for each seminal root separately using WinRHIZO and SmartRoot (https://smartroot.github.io/) software. The calculated root parameters included the length of the longest root, the number of seminal roots, the length of all seminal roots, the average diameter of seminal roots, the number of lateral roots, the length of all lateral roots, and the total root system length, surface, and volume. Statistical analyses were performed using ANOVA (*P* < 0.05) followed by Tukey’s honestly significant difference test (Tukey HSD test, *P* < 0.05).

### Analysis of Mitotic Activity

The Al treatment was performed as described in the previous section (“Aluminum Treatment in Hydroponics”). The mitotic activity of the meristematic root cells was analyzed in the *hvatr.g* and *hvatr.i* mutants and their wild-type parent after 7-day treatment with 10 μM Al^3+^. Additionally, for *hvatr.g*, the frequency of anaphases with chromosomal aberrations and the frequency of cells with micronuclei were estimated. Seedlings grown in the Magnavaca medium at pH 4.0 without Al^3+^ ions were used as a control. The roots were fixed in the ethanol/glacial acetic acid (3:1, *v*/*v*) solution and cytogenetic slides were prepared using the Feulgen’s squash technique. The experiment was carried out in three biological repetitions, with three plants per repetition. The cytogenetic parameters listed above were counted for minimum 10,000 cells for each *hvatr.g* experimental combination and for approximately 5,000 cells per *hvatr.i* experimental combination. Statistical analyses were performed using ANOVA (*P* < 0.05) followed by Tukey’s honestly significant difference test (Tukey HSD test, *P* < 0.05).

### Analysis of Cell Cycle Profile Using Flow Cytometry

The Al treatment of the *hvatr.g* mutant and cv. ‘Sebastian’ was performed as described in the previous section. Cell cycle analysis was performed for control roots and roots treated with 10 μM Al for 7 days. For one experimental replication, 20–30 root meristems were analyzed and three replications per treatment were used. The root tips were mechanically fragmented in a nuclei extraction buffer (CyStain^®^ UV Precise P, 05-5002, Sysmex) and the suspension of nuclei was filtered through a 30-μm nylon mesh in order to remove any debris and stained with a staining buffer (CyStain^®^ UV Precise P, 05-5002, Sysmex). Samples were analyzed with a CyFlow Space flow cytometer (Sysmex, Japan) with a 365-nm UV LED diode as the light source. The flow rate was adjusted to 20–40 nuclei per second. To determine the cell cycle phase, FloMax software with the Cell Cycle Analysis application was used.

### TUNEL Test

The terminal deoxynucleotidyl transferase-mediated dUTP nick-end labeling (TUNEL) test was used to detect and quantitatively analyze Al-induced DNA breaks in *hvatr.g*, *hvatr.i*, and ‘Sebastian’ roots. Al treatment was performed as described in the previous section. Control roots and roots treated with 5 and 10 μM Al for 7 days were fixed in 4% paraformaldehyde in phosphate-buffered saline (PBS) for 1 h at room temperature and then washed three times in PBS. Meristematic tissues were squashed in the PBS buffer. The prepared slides were frozen at −70°C. Prior to the TUNEL analysis, the slides were permeabilized in 0.1% Triton X-100 in 0.1% sodium citrate at 4°C for 2 min and rinsed in PBS. DNA fragment labeling was performed using a TUNEL reaction mixture (*in situ* Cell Death Detection Kit, Fluorescein, Roche) according to the provided protocol. The reaction containing an enzyme solution (terminal transferase) and a label solution (FITC-labeled nucleotides) in a 1:9 ratio (*v*/*v*) was applied to the preparations that were incubated at 37°C in the dark in a humid chamber. After 1 h, the preparations were rinsed three times in PBS and stained with DAPI (2 μg/ml) and then mounted in a Vectashield medium (Vector Laboratories). Preparations were examined with a Zeiss Axio Imager.Z.2 wide-field fluorescence microscope equipped with an AxioCam Mrm monochromatic camera (Zeiss, Germany). The frequency of TUNEL-positive FITC-labeled nuclei with DNA fragmentation was established based on analysis of 2,000 cells on two slides (each prepared from one root meristem) for the one repetition. For each experimental combination, two repetitions were analyzed. In total, 8,000 nuclei were analyzed for one combination. Statistical analyses were performed using Student’s *t* test with *P* < 0.05.

## Results

### Identification of Induced Mutations in the *HvATR* Gene

Seventeen mutations have been found in the two fragments of the *HvATR* gene screened using TILLING strategy (**Table 1**). The T1 amplicon (930 bp) encoding the UME domain (whose function has not yet been established) was analyzed in 6,144 M_2_ plants of the *Hor*TILLUS population. In total, five mutations were found within this fragment: three missense mutations (*hvatr.f*, *hvatr.g*, and *hvatr.i*) and two silent mutations. The mutation density calculated based on analysis of the T1 fragment was 1 mut./1,143 kb.

**Table 1:** Mutations identified in the *HvATR* gene.

*Allele*	Mutation position in gDNA (in CDS)	State of mutation in M_2_ plant	Type of mutation	Effect in protein	Mutated domain
*hvatr.a*	G11111A; G7524A	Homozygous	Silent	–	
*hvatr.b*	C11060T; C7473T	Heterozygous	Silent	–	
*hvatr.c*	G10792A; G7317A	Heterozygous	Silent	–	
***hvatr.d***	**C11104T; C7517T**	**Heterozygous**	**Missense**	**A2506V (alanine to valine)**	**PIKKc**
*hvatr.e*	C6023T; C3012T	Homozygous	Silent		
***hvatr.f***	**G5682A; G2767A**	**Homozygous**	**Missense**	**E923K (glutamic acid to lysine)**	**UME**
***hvatr.g***	**G6054A; G3043**	**Heterozygous**	**Missense**	**G1015S (glycine to serine)**	**UME**
*hvatr.h*	G6146A; G3135A	Homozygous	Silent	–	
***hvatr.i***	**G5623A; G2708A**	**Heterozygous**	**Missense**	**G903E (glycine to glutamic acid)**	**UME**
*hvatr.j*	G10693A; G7218A	Heterozygous	Silent	–	
*hvatr.k*	G10945A; -	Heterozygous	Intronic	–	
*hvatr.l*	G10486A; G7011A	Homozygous	Silent	–	
***hvatr.m***	**C10978T; C7391T**	**Heterozygous**	**Missense**	**A2464V** **(alanine to valine)**	**PIKKc**
*hvatr.n*	G10453A; G6978A	Homozygous	Silent	–	
***hvatr.o***	**C10574T; C7099T**	**Homozygous**	**Missense**	**L2367F (leucine to phenylalanine)**	**PIKKc**
***hvatr.p***	**T10604C; T7129C**	**Homozygous**	**Missense**	**W2377R (tryptophan to arginine)**	**PIKKc**
*hvatr.r*	C10871T;-	Heterozygous	Intronic	–	

The alleles that carry missense mutations are bolded.

The T2 amplicon (865 bp) encoding a fragment of the PIKKc domain responsible for kinase activity was screened in 6,189 M_2_ plants of the *Hor*TILLUS population. In total, 12 mutations were identified within this fragment. Among them, four were missense (*hvatr.d*, *hvatr.m*, *hvatr.o*, and *hvatr.p*), six were silent, and two occurred in the non-coding intron region. The mutation density calculated for the T2 fragment was 1 mut./446 kb, thus two times higher than in the UME domain-encoding fragment.

The mutations identified in the *HvATR* gene were confirmed by sequencing, and all of them, except for one (*hvatr.p*), were G/C to A/T transitions. Nine mutations were identified in the heterozygous state and eight mutations were in the homozygous state in M_2_ plants (**Table 1**).

Among all the mutations identified in this study, the missense mutations were chosen for functional analysis of the *HvATR* gene as they may affect protein activity and function. Mutants carrying the changes that led to the amino acid substitutions in the encoded protein were developed into homozygous lines and the M_3_ seed material was increased to M_4_ or, if needed, further generation to perform phenotyping and Al treatment experiments.

### The *hvatr.g* Mutant and Its Response to Aluminum

**Figure 1:**
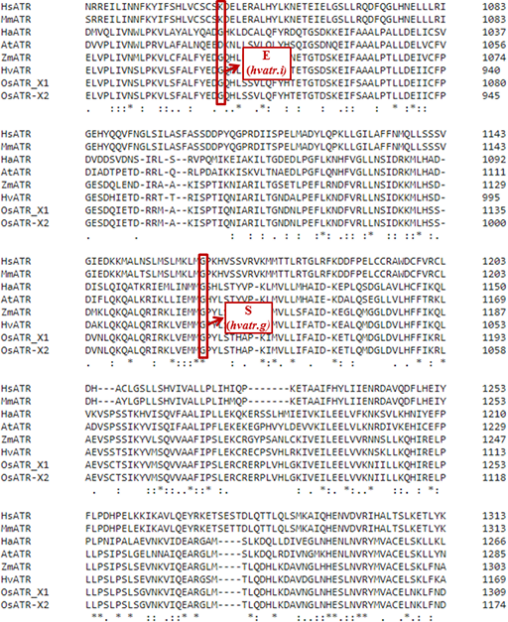
Multiple alignments of a fragment of the ATR protein sequences from various species, with the position of the substituted amino acids in the *hvatr.g* and *hvatr.i* mutants indicated by a *red frame*. *Hs, Homo sapiens*; *Mm, Mus musculus*; *Ha, Helianthus annuus*; *At, Arabidopsis thaliana*; *Zm, Zea mays*; *Hv, Hordeum vulgare*; *Os, Oryza sativa* (*X1* and *X2* represent two isoforms of this protein in rice). An * indicates positions which have a fully conserved residue. (A : indicates conservation between groups of strongly similar properties. A . indicates conservation between groups of weakly similar properties).

Of all the barley *atr* mutants that were preliminary tested for aluminum response, only one, *hvatr.g*, showed a strong Al-tolerant phenotype in all Al^3+^ concentrations used. The *hvatr.g* mutant carries a G6054A missense mutation in the UME domain that changes glycine-1015 to serine (G1015S) at the protein level. The multiple alignment of the ATR proteins from a broad spectrum of species showed that the glycine-1015 altered in the *hvatr.g* mutant is conserved among plants, animals, and humans (**Figure 1**). This suggests that the described mutation might have a significant impact on the protein function.

**Figure 2:**
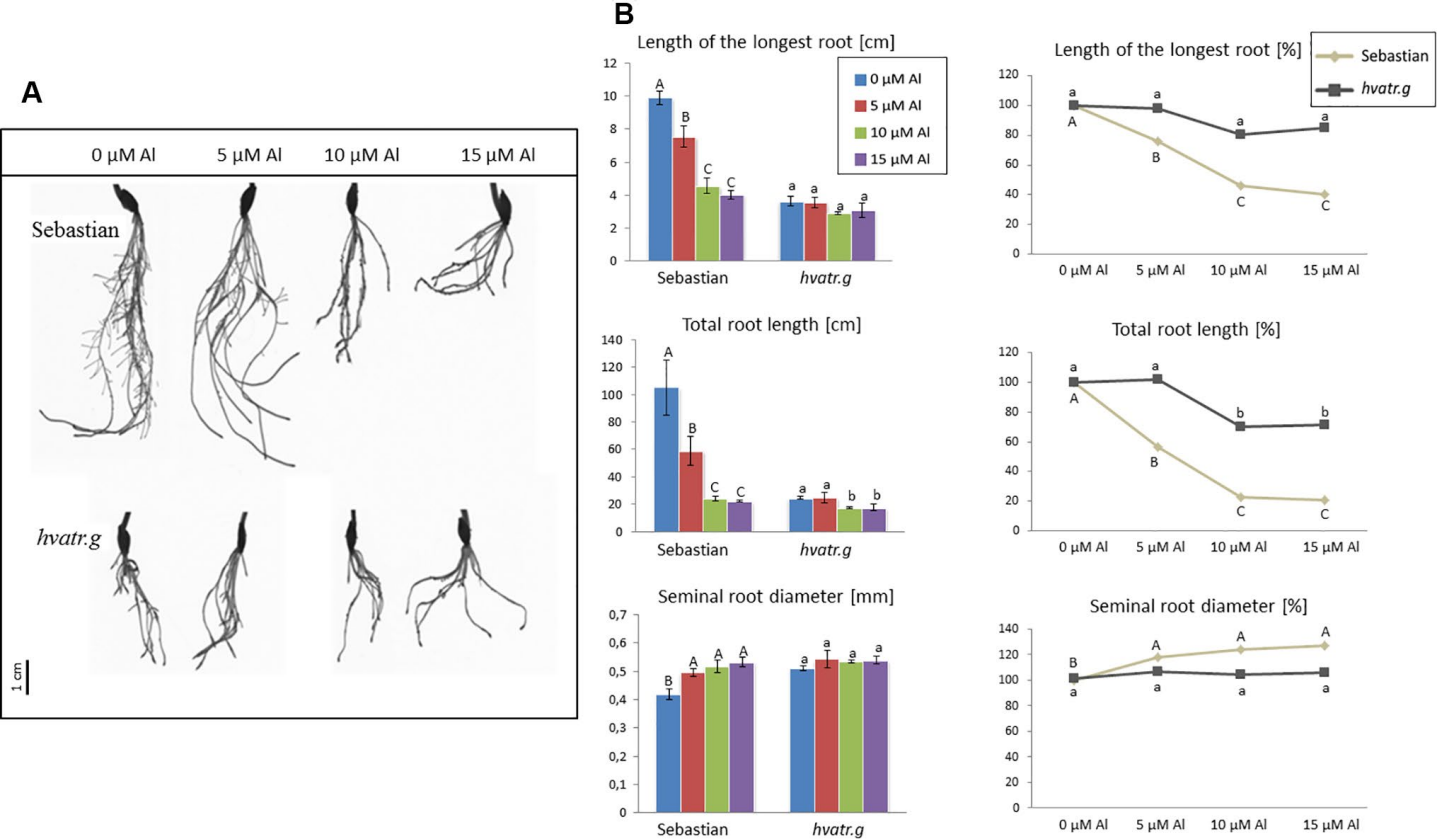
**(A)** Comparison of a root system of the *hvatr.g* mutant and cv. ‘Sebastian’ grown for 7 days in Magnavaca solution at pH 4.0 without Al and with 5, 10, and 15 μM of bioavailable Al^3+^ ions. **(B)** Main root parameters of ‘Sebastian’ and the *hvatr.g* mutant grown for 7 days in Magnavaca solution at pH 4.0 without Al and with 5, 10, and 15 μM of bioavailable Al^3+^ ions. The parameters are presented as direct values (in centimeters) and as percentage, where 100% is the value for untreated roots of the genotype. Presented parameters: length of the longest root, total length of the whole root system (seminals + laterals), and average diameter of seminal roots. Statistically significant differences between different doses of Al within each genotype were assessed using ANOVA (*P* < 0.05) followed by Tukey’s honestly significant difference test (Tukey HSD test, *P* < 0.05) and are indicated by different uppercase letters for the ‘Sebastian’ variety and by different lowercase letters for the *hvatr.g* mutant.

Detailed evaluation of root system growth after aluminum treatment was performed for the *hvatr.g* mutant and its parent variety ‘Sebastian’ grown in the Magnavaca solution without Al and supplemented with 5, 10, and 15 μM Al. The *hvatr.g* mutant was characterized by a shorter root system than the wild type in the control combination; however, the growth of its roots was not affected by Al, contrary to ‘Sebastian’ roots (**Figures 2A, B**). The length of the longest root of ‘Sebastian’ was reduced by 25%, 55%, and 60% in 5, 10, and 15 μM Al, respectively, whereas the length of the longest root of the mutant was not reduced in any of the Al doses tested (**Figure 2B**). The total root length that is the sum of the length of all seminals and laterals was significantly reduced in ‘Sebastian’ in all Al concentrations (up to 80%), while in the mutant it was reduced only in the higher Al concentrations and only by approx. 30% (**Figure 2B**). Aluminum caused an increase of the seminal root diameter in ‘Sebastian’ (which is a typical symptom of Al toxicity), whereas in the mutant the root diameter was not affected by any of the Al concentrations used (**Figure 2B**). As a result, the surface and volume of the whole root system of ‘Sebastian’ were significantly reduced, up to 70% and 50%, respectively, while these parameters in the *hvatr.g* mutant were reduced only to a small extent, up to 20% (data not shown).

### Detailed Analysis of Root System Architecture in the *hvatr.g* Mutant

**Figure 3:**
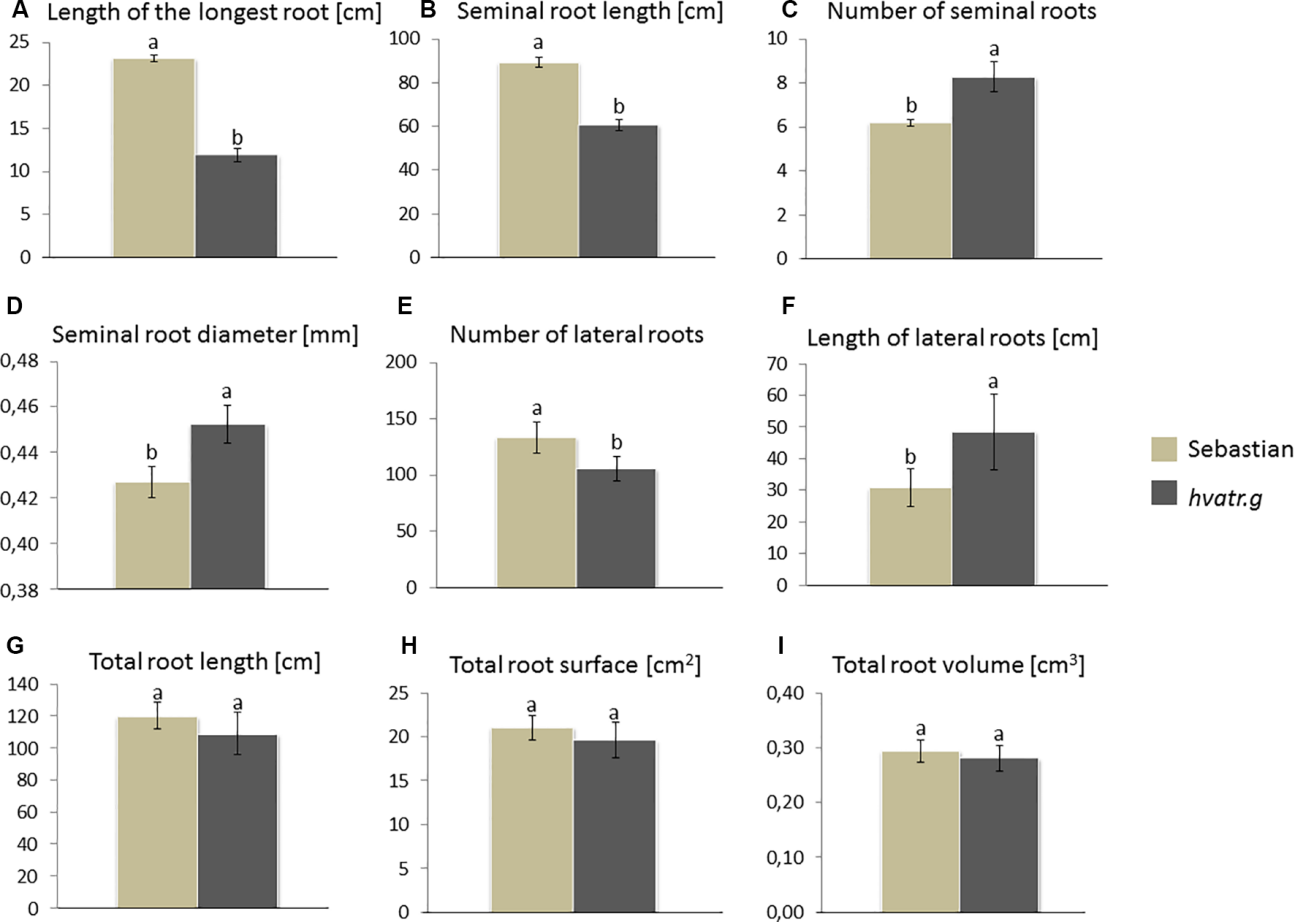
Main root parameters of ‘Sebastian’ and the *hvatr.g* mutant grown for 2 weeks in a flood-and-drain semi-hydroponics irrigated with a full-strength Hoagland solution. **(A)** Length of the longest root. **(B)** Length of all seminal roots. **(C)** Number of seminal roots. **(D)** Average diameter of seminal roots. **(E)** Number of lateral roots. **(F)** Length of all lateral roots. **(G)** Length of the whole root system (seminals + laterals). **(H)** Surface of the whole root system (seminals + laterals). **(I)** Volume of the whole root system (seminals + laterals). Statistical analyses were performed using ANOVA (*P* < 0.05) followed by Tukey’s honestly significant difference test (Tukey HSD test, *P* < 0.05) to assess the differences between two genotypes. Statistically significant differences are indicated by different letters.

Due to the fact that the root system of the *hvatr.g* mutant was Al-tolerant, but significantly reduced, compared to the wild type, after 7 days of growth in a hydroponic culture in Magnavaca solution at pH 4.0 (control in the Al experiment), we decided to analyze the mutant root phenotype after a longer growth period in more optimal conditions. The seedlings of the mutant and the wild type were grown for 14 days in a flood-and-drain semi-hydroponic system and were irrigated with a full-strength Hoagland solution (pH ∼6.0), rich in nutrients. We observed that also under these optimal conditions, the seminal roots of the *hvatr.g* were in general shorter than in the case of ‘Sebastian’ and the length of the longest root was even twice reduced (11 and 23 cm for the mutant and the wild type, respectively; **Figure 3**). However the *hvatr.g* mutant produced more seminal roots (eight and six for the mutant and the wild type, respectively), and they were characterized by an increased diameter. Moreover, *hvatr.g* developed longer lateral roots (the total lateral root length was significantly higher compared to ‘Sebastian’), which all resulted in no difference of the length, surface, and volume of the whole root system between the mutant and its wild type (**Figure 3**).

### Influence of Al Treatment on the Mitotic Index and Cell Cycle Profile of the *hvatr.g* Mutant

**Figure 4:**
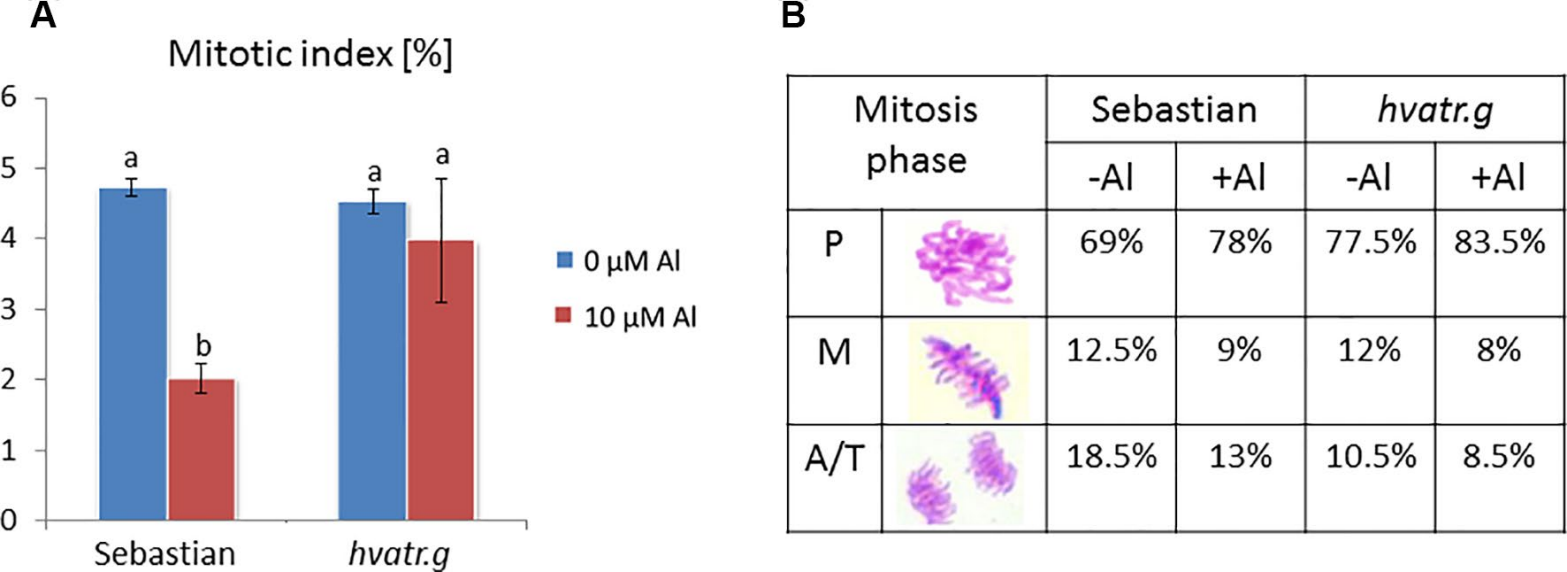
Cytological effects of Al in the root cells of cv. ‘Sebastian’ and the *hvatr.g* mutant. **(A)** Mitotic activity in the root meristems of untreated and Al-treated plants. **(B)** Frequency of mitosis phases (prophases, metaphases, and anaphases/telophases). Statistical analyses were performed using ANOVA (*P* < 0.05) followed by Tukey’s honestly significant difference test (Tukey HSD test, *P* < 0.05). Statistically significant differences are indicated by different letters.

We have analyzed the mitotic activity of the meristematic root cells of the *hvatr.g* mutant and its wild type after 1 week of 10 μM Al treatment. The mitotic indices in the root meristems of both ‘Sebastian’ and *hvatr.g* mutant were at the same level, approx. 4.5% in control conditions (Magnavaca solution, pH 4.0). Aluminum treatment caused the reduction of the mitotic index in ‘Sebastian’ to 2%, whereas it did not affect the mutant (**Figure 4A**). The frequencies of the particular mitotic phases were calculated and showed that the majority of the dividing cells were in the prophase stage; however, in the mutant, this fraction of mitotically active cells was higher than in the wild type (77.5% and 69%, for *hvatr.g* and ‘Sebastian’, respectively). Aluminum treatment increased slightly the percentage of prophase cells and decreased the number of cells in the other phases of mitosis (metaphases and anaphases/telophases) in both genotypes (**Figure 4B**).

**Figure 5:**
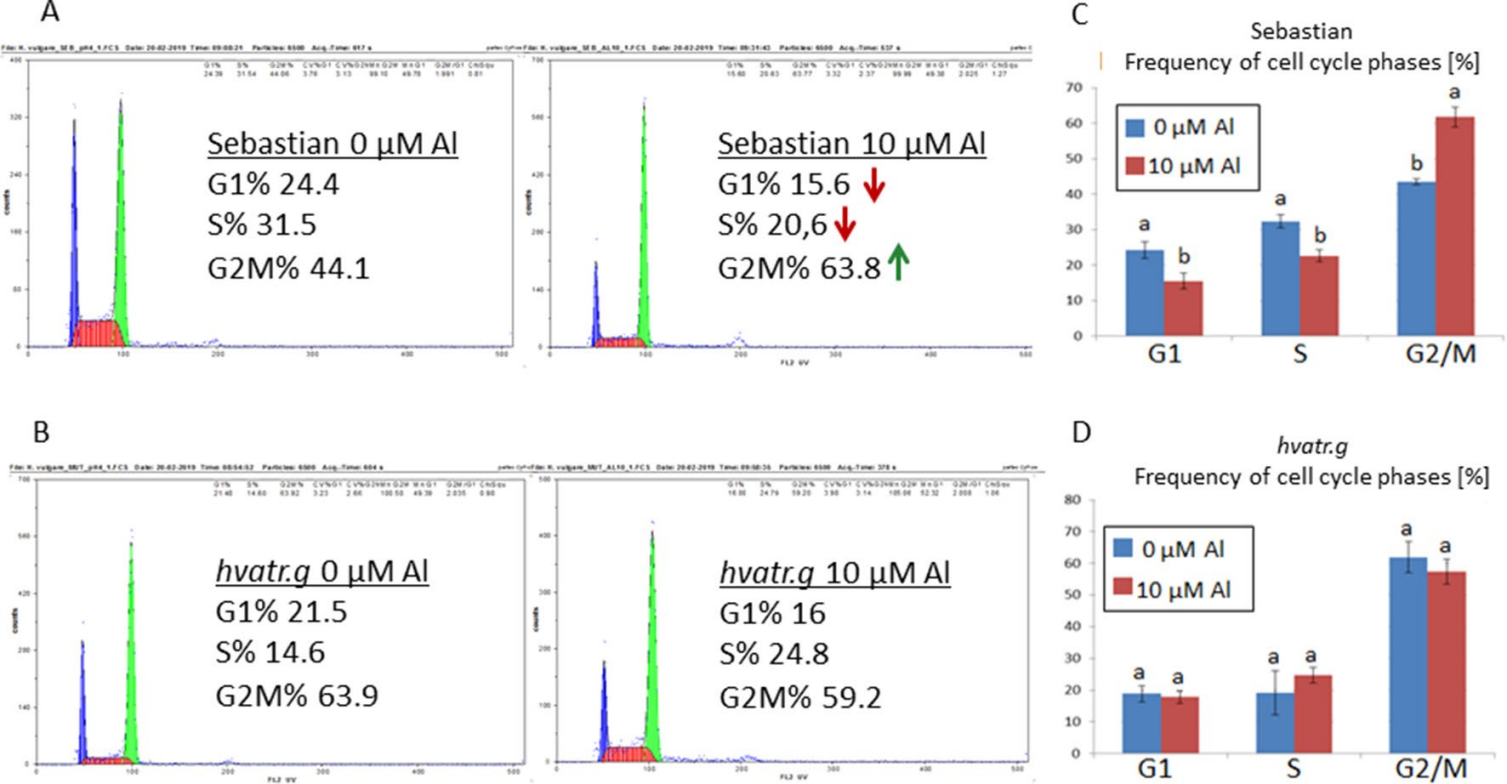
Flow cytometric analysis of the cell cycle profiles in root meristematic cells of ‘Sebastian’ and the *hvatr.g* mutant. **(A)** Examples of representative histograms for ‘Sebastian’ grown in control conditions and treated with 10 μM Al. **(B)** Examples of representative histograms for the *hvatr.g* mutant grown in control conditions and treated with 10 μM Al. **(C)** Frequencies of cell cycle phases in the root meristems of ‘Sebastian’ untreated and treated with 10 μM Al. **(D)** Frequencies of cell cycle phases in the root meristems of the *hvatr.g* mutant untreated and treated with 10 μM Al. Statistical analyses were performed using ANOVA (*P* < 0.05) followed by Tukey’s honestly significant difference test (Tukey HSD test, *P* < 0.05) in order to assess the differences between treated and untreated samples. Statistically significant differences are indicated by different letters.

Analysis with the use of flow cytometry was performed in order to check the effect of Al treatment on the cell cycle in the roots of the analyzed genotypes. The cell cycle profile in control conditions differed between the *hvatr.g* mutant and its wild type. The mutant was characterized by a higher frequency of cells in the G2/M phase (61%) and a lower frequency of cells in the S phase (20%) than the wild type (43% and 32% for the G2/M and S phases, respectively). The aluminum treatment did not change the cell cycle profile in the *hvatr.g* mutant, while it caused the decrease of the cells in the S phase (to 22%) and the increase of cells in the G2/M phase (to 62%) in ‘Sebastian’ (**Figure 5**).

### Assessment of DNA Damage Induced by Aluminum in the *hvatr.g* Mutant

**Figure 6:**
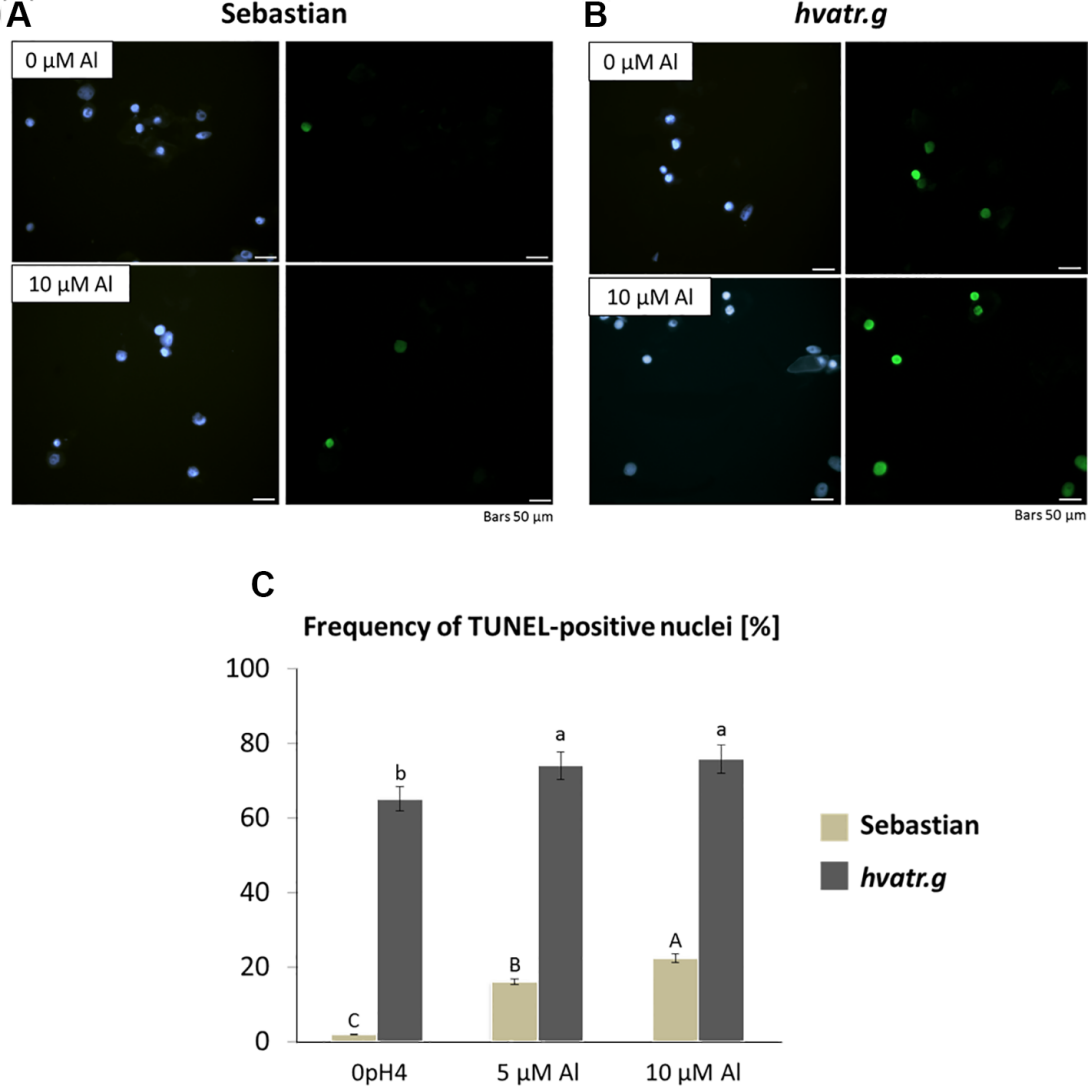
Results of the TUNEL test in the root meristematic cells of ‘Sebastian’ and the *hvatr.g* seedlings treated with Al. **(A)** Examples of damaged nuclei observed in control ‘Sebastian’ roots and roots treated with 10 µM Al. Left Images of DAPI-stained nuclei. Right Images from the FITC channel. **(B)** Examples of damaged nuclei observed in control *hvatr.g* roots and roots treated with 10 µM Al. Left Images of DAPI-stained nuclei. Right Images from the FITC channel. **(C)** Frequency of labelled nuclei in the root cells of analyzed genotypes treated with 5 and 10 µM Al. The significant differences (P < 0.05) between the groups are indicated by different letters (uppercase letters for 'Sebastian' and lowercase letters for *hvatr.g* mutant).

The TUNEL test was applied in order to analyze the frequency of nuclei with DNA breaks in the root meristems of the *hvatr.g* mutant and its wild-type parent after 7 days of treatment with 5 and 10 μM Al. To determine the percentage of damaged nuclei, all cells were simultaneously stained with DAPI. The nuclei that had a green fluorescence detected in the FITC channel were characterized by DNA damage (**Figures 6A, B**). The analysis revealed that as much as 60% of the *hvatr.g* mutant nuclei showed TUNEL-specific fluorescence in the control conditions, while the parent variety ‘Sebastian’ in the control hydroponics had 1.9% of TUNEL-positive nuclei. Treatment with 5 or 10 μM Al significantly increased the frequency of damaged nuclei: by about 10% in the mutant, whereas by 14% and 20% in ‘Sebastian’ root cells, respectively (**Figure 6C**).

Additionally, we have calculated the frequency of cells with micronuclei, which arise from unrepaired double-strand DNA breaks, as well as the frequency of chromosome aberrations during anaphase. Similar to the results of the TUNEL test, the *hvatr.g* mutant showed a five times higher frequency of cells with micronuclei under control conditions than the wild type. Al treatment increased the number of such cells in both genotypes, almost twice in the mutant and five times in the wild-type parent (**Supplementary Material 3**). Correspondingly, under control conditions, the chromosomal aberration index was also higher in the mutant compared to the wild-type parent, and it increased significantly after Al treatment in both genotypes (**Supplementary Material 4**).

### Analysis of the *hvatr.i* Mutant Carrying Another Mutation in the *HvATR* Gene

The *hvatr.g* mutant was induced by chemical mutagenesis; therefore, it might carry other mutations affecting the traits analyzed in this study. In order to confirm that the Al-tolerant phenotype of the *hvatr.g* mutant (described in the previous sections) was indeed caused by the identified G6043A mutation in the *HvATR* gene and not by other mutations present in the *hvatr.g* genome, we screened all other TILLING mutants carrying different missense mutations in the analyzed gene for their response to Al treatment. We found that one of these mutants, *hvatr.i*, showed a similar to *hvatr.g*, but a weaker Al-tolerant phenotype. The *hvatr.i* mutant carries a G5623A transition leading to the change of glycine-903 to glutamic acid (G903E) in the UME domain (**Figure 1**).

**Figure 7:**
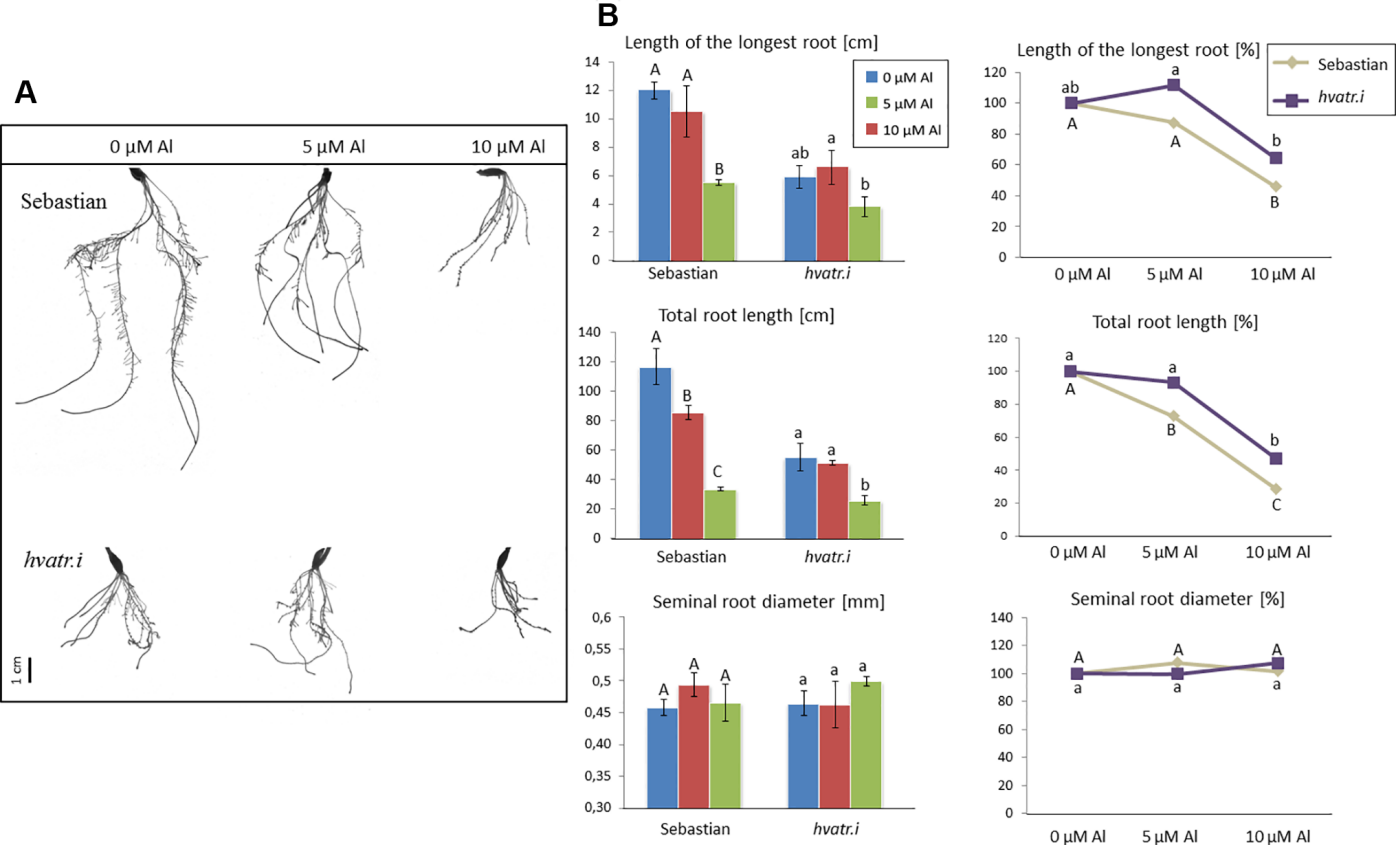
**(A)** Comparison of the root system of the *hvatr.i* mutant and cv. ‘Sebastian’ grown for 7 days in Magnavaca solution at pH 4.0 without Al and with 5 and 10 μM of bioavailable Al^3+^ ions. **(B)** Main root parameters of ‘Sebastian’ and the *hvatr.i* mutant grown for 7 days in Magnavaca solution at pH 4.0 without Al and with 5 and 10 μM of bioavailable Al^3+^ ions. The parameters are presented as direct values (in centimeters) and as percentage, where 100% is the value for untreated roots of the genotype. Presented parameters: length of the longest root, total length of the whole root system (seminals + laterals), and average diameter of seminal roots. Statistically significant differences between different doses of Al within each genotype were assessed using ANOVA (*P* < 0.05) followed by Tukey’s honestly significant difference test (Tukey HSD test, *P* < 0.05) and are indicated by different uppercase letters for the ‘Sebastian’ variety and by different lowercase letters for the *hvatr.i* mutant.

Evaluation of *hvatr.i* root system growth after aluminum treatment was performed in the same way as described for the *hvatr.g* mutant. Two concentrations of Al were tested: 5 and 10 μM Al^3+^. The *hvatr.i* mutant, similarly to *hvatr.g*, was characterized by a shorter root system than the wild type when grown in the control medium without Al; however, the growth of its roots was much less affected by Al treatment than the growth of ‘Sebastian’ roots (**Figures 7A, B**). The length of the longest root of ‘Sebastian’ was reduced by 12.5% and 54% after treatment with 5 and 10 μM Al, respectively, whereas the length of the longest root of the *hvatr.i* mutant was not affected (or even slightly increased) by 5 µM Al and was reduced by 36% in the medium with 10 μM Al (**Figure 7B**). The total root length of all seminals and laterals was significantly reduced in ‘Sebastian’ in both Al concentrations tested (by 27% and 72% in 5 and 10 μM Al, respectively), while in the *hvatr.i* mutant it was also reduced, but to a lesser extent (by 7% and 53% in 5 and 10 μM Al, respectively; **Figure 7B**). In this experiment, we have not observed any differences in the average seminal root diameter between the analyzed genotypes.

**Figure 8:**
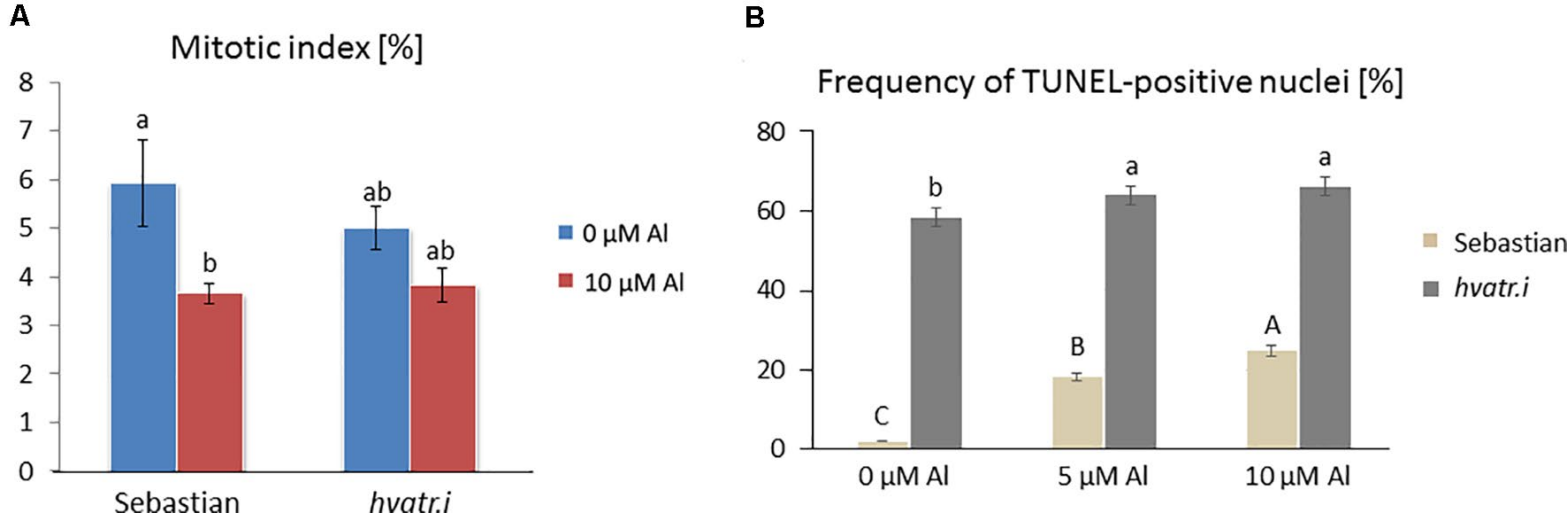
Cytological analysis of the *hvatr.i* mutant and its parent variety ‘Sebastian’. **(A)** Mitotic activity in the root meristems of untreated and Al-treated plants. **(B)** Results of the TUNEL test—frequency of labeled nuclei in the root cells of analyzed genotypes untreated and treated with 5 and 10 µM Al. The significant differences (P < 0.05) between the groups are indicated by different letters (uppercase letters for 'Sebastian' and lowercase letters for *hvatr.g* mutant).

We have analyzed the mitotic activity of the root cells in the meristematic zone of *hvatr.i* seedlings grown in the medium with 10 µM Al and without Al at pH 4.0. The preliminary analysis revealed that in the wild-type cv. ‘Sebastian’, the mitotic index was reduced by 40% after Al treatment, whereas in the analyzed mutant the frequency of dividing cells was reduced by 24% (**Figure 8A**).

To assess the level of DNA damage caused by Al, the TUNEL test was applied. Similarly to the *hvatr.g* mutant, the percentage of TUNEL-positive nuclei in the *hvatr.i* mutant was much higher than in ‘Sebastian’, even under control conditions, and it was further increased by aluminum treatment. Almost 60% of the *hvatr.i* mutant nuclei was damaged in the control medium (at pH 4.0); however, their fluorescence was lower than that in the *hvatr.g* mutant. Under the same control conditions, only 2% of ‘Sebastian’ nuclei were TUNEL-positive. Treatment with 5 and 10 μM Al significantly increased the frequency of damaged nuclei in both genotypes, but to a much lesser extent in the *hvatr.i* than in the parent cultivar: by about 6% and 8% in the mutant, whereas by 15% and 22% in ‘Sebastian’ root cells, respectively (**Figure 8B**). All these observations indicate that the *hvatr.i* mutant expresses similar to *hvatr.g*, though a weaker phenotype in regard to the Al response and DNA damage repair.

## Discussion

In essence, activation of the DDR pathway in response to DNA damage leads to cell cycle stoppage, the activation of DNA repair processes, and to programmed cell death (reviewed in Kim et al., 2019). We have identified mutants, named *hvatr.g* and *hvatr.i*, that carry missense mutations in the *HvATR* gene. ATR is a serine/threonine kinase that acts as a central regulator in the DDR pathway. Together with another kinase, ATM, it is responsible for the activation of this pathway and transduction of a signal in response to DNA damage (Culligan et al., 2004). The mutations identified in our TILLING mutants lead to the substitution of very conserved amino acids, glycine-1015 to serine in the *hvatr.g* mutant and glycine-903 to glutamic acid in the *hvatr.i* mutant. Both altered amino acids are within the UME domain of the ATR protein. This domain has not yet been functionally characterized; however, it is predicted to be required for protein–protein interactions (Rounds and Larsen, 2008).

Here, we present that both mutants, *hvatr.g* and *hvatr.i*, are impaired in the DDR pathway. They accumulated DNA damages in control hydroponic conditions, when seedlings were grown in the Magnavaca medium at pH 4.0. The damages have been proven by TUNEL analysis, which revealed that approx. 60% of both mutants’ nuclei possessed DNA nicks and breaks, whereas in the wild type only approx. 2% of nuclei were TUNEL-positive. Additionally, in the *hvatr.g* mutant, which was characterized in more detail, the frequencies of micronuclei and chromosome aberrations were statistically higher in the mutant than in its wild-type parent under control conditions. The work performed on *Arabidopsis* has also shown, based on a comet assay, that the *atr* mutant (in the *als3-1* background) was characterized by a higher DNA damage level in control conditions than Col-0; however, this difference was not that substantial (Rounds and Larsen, 2008). Interestingly, even though the frequency of dividing cells was not altered in the *hvatr.g* mutant, the cell cycle profile differed from that of ‘Sebastian’. The mutant possessed fewer cells in the S phase and more cells in the G2/M phase. Moreover, the cells in the stage of prophase represented a greater percentage of dividing cells in the mutant than in ‘Sebastian’. Taken together, these results show that the transduction of a signal of DNA damage does not function properly in the *hvatr.g* mutant, confirming the crucial role of ATR in the DDR pathway in barley.

Aluminum toxicity is considered as one of the most important agricultural problems worldwide. The first evidence that the activation of the DDR pathway is, at least in part, responsible for Al-induced root growth inhibition came from studies on *Arabidopsis* (summarized in Eekhout et al., 2017). Here, we confront this knowledge with an agronomically important species and present the evidence that the change in the DDR pathway may affect response to aluminum in barley.

Our *hvatr.g* and *hvatr.i* mutants manifested Al-tolerant phenotypes. The longest seminal root of *hvatr.g* was not reduced even by the highest aluminum concentration applied, whereas the root system of the cultivar ‘Sebastian’ was severely reduced in all Al concentrations tested. These results are consistent with the work performed on *Arabidopsis*, where analysis of the root growth of *atr* mutants showed a high level of Al tolerance. One of the described *Arabidopsis* mutants possessed mutation within the UME domain, which confirms that this domain is necessary for proper activity of the ATR protein (Rounds and Larsen, 2008).

In our study, the reduction of root length in the wild-type cv. ‘Sebastian’ after exposure to Al might result, at least in part, from the decreased mitotic activity in the root meristems and the increased level of DNA damage. Our previous work performed for the same genotype treated with aluminum in Hoagland solution has shown that Al treatment significantly reduced the mitotic activity of the root tip cells (Jaskowiak et al., 2018). Similar results were obtained in Al studies of other species, for example *Helianthus annuus* (Kumar and Srivastava, 2006; Li et al., 2015), *Vicia faba* (Zhang et al., 2018), and *Allium cepa* (Qin et al., 2010). Nonetheless, the root meristematic cells of the barley *hvatr.g* mutant studied here were dividing despite the presence of this genotoxic agent in the medium, which further increased the level of DNA damage observed in control conditions. The frequency of dividing cells has not been changed after Al treatment. The same tendency was observed in the *hvatr.i* mutant—although the frequency of dividing cells decreased after Al treatment, this reduction was not that high as in ‘Sebastian’. Moreover, the cell cycle profile of the *hvatr.g* mutant was not affected by Al treatment, whereas in the wild type the aluminum treatment caused an increase in the number of cells arrested in the G2 phase. Similarly, the inhibition of root growth caused by Al in *Arabidopsis* correlated with the cell cycle arrest in the G2 stage, which was accompanied by the accumulation of Cyclin B1;1 in the root tips of the wild type and Al-hypersensitive *als3-1* mutant. In the *atr* mutant, no concomitant increase in the activity of *CycB1;1* was observed, which indicated that cell division was not arrested at the G2 stage (Rounds and Larsen, 2008). Correspondingly, in another *Arabidopsis* Al-hypersensitive mutant, *star1* (with T-DNA insertion in *STAR1* encoding a nucleotide binding domain of ABC transporter), the expression of *CycB1;1* dramatically increased in root meristems after exposure to even low doses of Al, which did not cause inhibition of root growth in the wild type. This suggests that the cell cycle progression was halted in *star1* in response to the low level of Al (Zhang et al., 2018).

The DNA-damaging effect of Al was observed in our study for all genotypes tested. Using TUNEL, micronuclei and chromosome aberration tests, we demonstrated the increase in the frequency of root tip cells carrying DNA damages after Al treatment. Our data support other studies showing that aluminum has an impact on DNA integrity, probably through a direct binding to the DNA phosphate backbone (Silva et al., 2000; Jaskowiak et al., 2018). Studies performed in several Gramineae species showed DNA fragmentation after Al treatment in rye, barley, and oat roots, but not in maize and wheat (Vardar et al., 2016). These observations indicate that plants differ in response to phytotoxic Al^3+^ ions. Barley is known to be the most sensitive to Al among cereals; however, it shows some genotype variation (Ma et al., 2004; Ma et al., 2016), which makes it possible to breed more Al-tolerant cultivars. Our barley *atr* mutants with the impaired DDR pathway may serve as a tool to study Al tolerance in this important crop species. It should be noted that they are fully fertile, which indicates that the mutants do not carry DNA damages in generative cell lines, where the DNA lesions might be potentially repaired through a different pathway (not ATR-dependent).

The fact that TILLING mutants possess a high number of mutations in their genomes might be a limitation in using them for functional gene studies. However, it should be noted that a vast majority of mutations after classical chemical mutagenesis occur in non-coding regions (Kurowska et al., 2012). The barley genome is very large (approx. 5.3 Gbp) and coding sequences constitute only 1.3% of its size (Mascher et al., 2017). Moreover, many mutations in the coding sequences are silent or do not affect the protein function. Therefore, the probability of obtaining knockdown or knockout mutations in genes involved in the same process of interest (for example, DNA repair through the DDR pathway) in the same mutated plant is very low (Szurman-Zubrzycka et al., 2018). Nevertheless, here, we described two independent TILLING mutants carrying different mutations in the *HvATR* gene. These mutants showed similar phenotypes related to aluminum response and DNA repair, which confirms that, indeed, the disruption of ATR function is responsible for the observed alterations and that these mutants, together, are a useful tool for functional analysis of the *ATR* gene in barley.

## Conclusions

We have identified barley TILLING mutants, *hvatr.g* and *hvatr.i*, carrying different missense mutations in the *HvATR* gene that showed an impaired repair of DNA lesions, but the Al-tolerant phenotype. The high frequency of DNA damages observed in the mutants already in the control conditions did not lead to the inhibition of cell cycle progression. Al treatment increased the level of DNA damages, but did not affect the mitotic activity and the cell cycle profile in the *hvatr.g* mutant. The *hvatr.i* mutant showed a similar, although a weaker, Al-tolerant phenotype. We demonstrate that ATR is required for detection of DNA damage caused by toxic Al^3+^ ions in barley. We conclude that the reduction of root growth in response to aluminum is, at least in part, triggered by the ATR-dependent activation of DDR response leading to the arrest of cell cycle. The identified *hvatr.g* and *hvatr.i* mutants may serve as a useful tool in further studies on the DDR pathway in cereal species.

## Data Availability Statement

The raw data supporting the conclusions of this manuscript will be made available by the authors, without undue reservation, to any qualified researcher.

## Author Contributions

IS conceived and supervised the project. MS-Z and JJ identified TILLING mutants. MS-Z, MN and MD conducted hydroponic experiments and analyzed the root system. MS-Z and MD performed cytological analysis. JK performed TUNEL analysis. MS-Z and IS analyzed the data and wrote the manuscript.

## Funding

This work was supported by grant from ERA-NET for Coordinating Plant Sciences. The authors gratefully acknowledge financial support of the research by the National Centre for Research and Development (grant ERA-CAPS-II/2/2015).

## Conflict of Interest

The authors declare that the research was conducted in the absence of any commercial or financial relationships that could be construed as a potential conflict of interest.
