# Supplementary material for: ATR, a DNA Damage Signaling Kinase, Is Involved in Aluminum Response in Barley
Source: Front Plant Sci. 2019 Oct 22;10:1299. doi: 10.3389/fpls.2019.01299 (PMC6817586; doi:10.3389/fpls.2019.01299)
Supplement: Supplementary file 1 [file Table_1.docx]

Supplementary Material 1


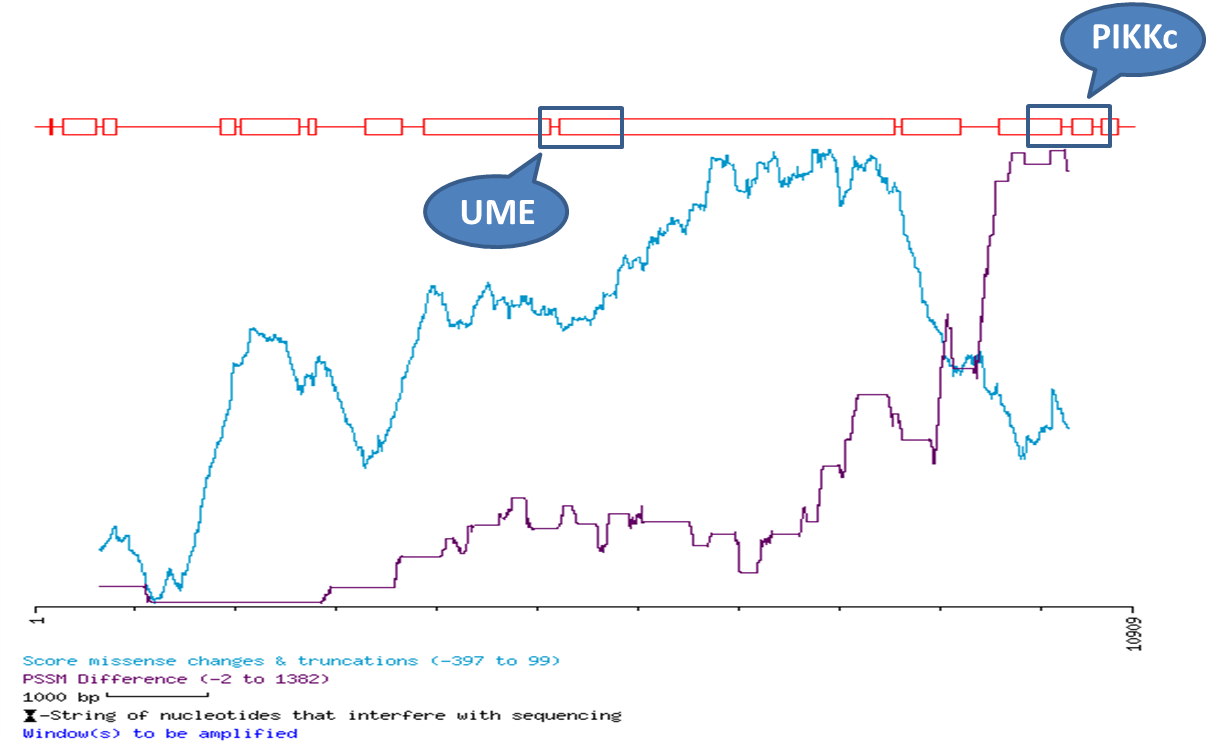


**Supplementary Figure 1.** CODDLE analysis (Codons Optimized to Discover Deleterious Lesions; <http://blocks.fhcrc.org/~proweb/input/>) showing the structure and the most conserved fragments of *HvATR* gene (blue line – score missense changes and truncations; violet – the PSSM difference). T1 and T2 amplicons are indicated in blue frames. T1 amplicon encodes UME domain and T2 amplicon encodes a part of PIKKc domain.
