# Supplementary material for: ATR, a DNA Damage Signaling Kinase, Is Involved in Aluminum Response in Barley
Source: Front Plant Sci. 2019 Oct 22;10:1299. doi: 10.3389/fpls.2019.01299 (PMC6817586; doi:10.3389/fpls.2019.01299)
Supplement: Supplementary file 2 [file Table_2.docx]

Supplementary Material 2

The sequences of primers used for amplification of T1 amplicon:

UME_F: 5’ – CTGCATGAACTGGCAAACCA – 3’

UME_R: 5’ – AAGACCATCAACGGCATCCTT – 3’

The sequences of primers used for amplification of T2 amplicon:

PIKKc_F : 5’ – TTGCCGTGGTTCCACTTACAGAA – 3’

PIKKc_R: 5’ – TGGATCATCGTTAGGAAACACG – 3’

The PCR reaction mix (T1 and T2 amplicon):

| ddH_2_O | 13.9 μl |
| --- | --- |
| 10 x buffer B (EURx) | 2 μl |
| dNTPs (5mM) (Promega) | 0.8 μl |
| Primer F* (20 pmol/μl) | 0.4 μl |
| Primer R** (20 pmol/μl) | 0.4 μl |
| ColorTaq polymerase (EURx) | 0.5 μl |
| DNA (100 ng/μl) | 2 μl |

*mix of IRDye-700 labeled and unlabeled primers (3:2)

**mix of IRDye-800 labeled and unlabeled primers (4:1)

Temperature profile of PCR reaction (T1 amplicon):

| Initial denaturation | 95°C | 5 min |
| --- | --- | --- |
| Denaturation | 95°C | 30 sec  x 40 cycles |
| Annealing | X*°C | 30 sec |
| Elongation | 72°C | 1 min 30 sec |
| Final elongation | 72°C | 5 min |
| Pause | 4°C | ∞ |

*65°C for T1 amplicon; 63°C for T2 amplicon
