# Supplementary material for: ATR, a DNA Damage Signaling Kinase, Is Involved in Aluminum Response in Barley
Source: Front Plant Sci. 2019 Oct 22;10:1299. doi: 10.3389/fpls.2019.01299 (PMC6817586; doi:10.3389/fpls.2019.01299)
Supplement: Supplementary file 3 [file Table_3.docx]

Supplementary Material 3


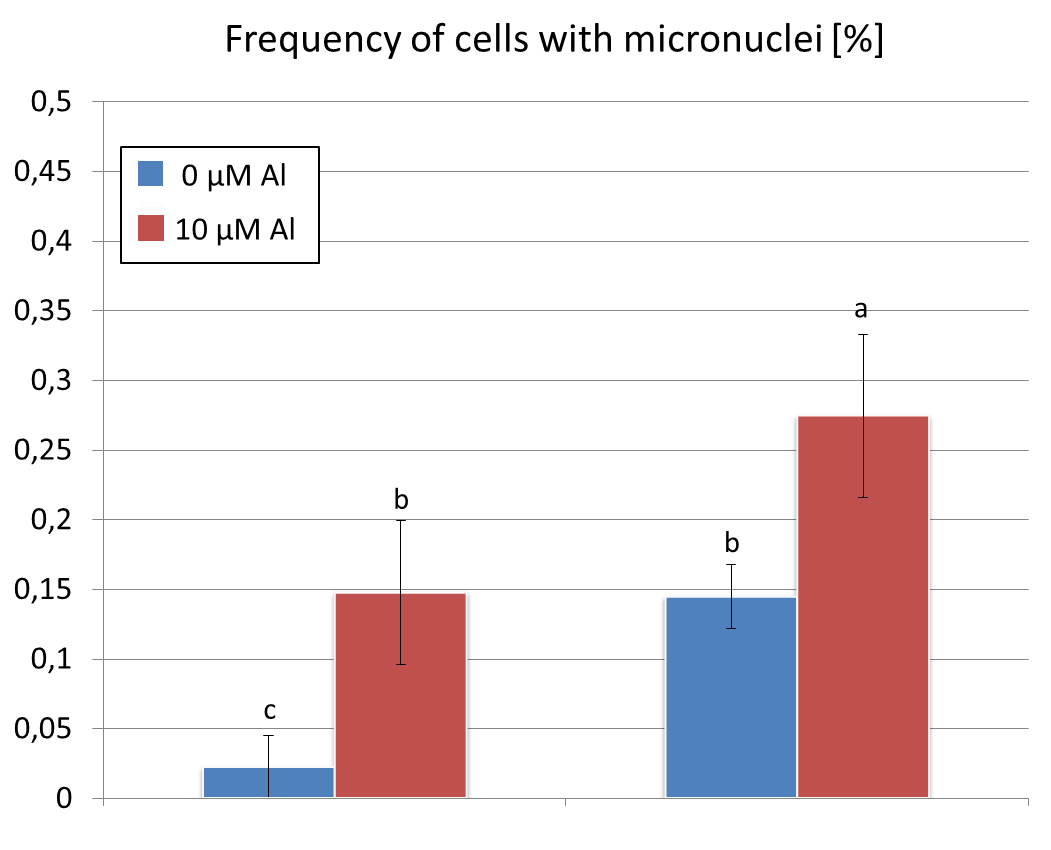


Sebastian *hvatr.g*

**Supplementary Figure 3.** The frequency of cells with micronuclei observed in root meristems of cv. ‘Sebastian’ and *hvatr.g* mutant untreated and treated with 10 μM Al. Statistically significant differences are indicated by different letters.
